# Evaluation of the cytotoxic and genotoxic effects by melamine and cyanuric acid co-exposure in human embryonic kidney 293 cells

**DOI:** 10.1590/1414-431X20209331

**Published:** 2020-04-27

**Authors:** Xianrong Xu, Jing Lu, Hongqiang Sheng, Long Zhang, Tieer Gan, Jianyun Zhang, Yuying Xu, Xinqiang Zhu, Jun Yang

**Affiliations:** 1 Department of Prevention Medicine, Hangzhou Normal University School of Medicine, Hangzhou, Zhejiang, China; 2 Zhejiang-California International Nanosystems Institute, Hangzhou, Zhejiang, China; 3 Department of Basic Medicine, Zhejiang University School of Medicine, Hangzhou, Zhejiang, China; 4 Hospital Infection Control Department, Zhejiang Provincial TCM Hospital, Hangzhou, Zhejiang, China; 5 Department of Toxicology, Zhejiang University School of Public Health, Hangzhou, Zhejiang, China; 6 Zhejiang Provincial Center for Uterine Cancer Diagnosis and Therapy Research, The Affiliated Women's Hospital, Zhejiang University, Hangzhou, Zhejiang, China

**Keywords:** Melamine, Cyanuric acid, γH2AX, DNA damage, Genotoxicity

## Abstract

The melamine and cyanuric acid (CA) complex has been suggested to cause the toxic effects observed in melamine-contaminated food or milk. However, the cytotoxic and genotoxic effects of co-exposure to melamine and CA are not fully clear. Therefore, the cytotoxic effects of melamine and CA were first examined by co‐exposure in human kidney 293 cells using the MTT assay. During a 24-h period for the three concentrations tested (0.5, 1, and 5 mg/mL), neither melamine nor CA alone showed significant toxic effects on 293 cells at 0.5 mg/mL, while higher concentrations led to decreased in cell viability. However, co-exposure to several combinations of melamine and CA [100:1, 10:1, 1:10, and 1:100 (v:v), at a final concentration of 0.5 mg/mL] did cause cytotoxicity with higher levels of CA leading to higher cytotoxicity. By contrast, while neither melamine nor CA alone induced phosphorylated-H2AX (γH2AX) foci formation, melamine and CA at a 100:1 ratio induced γH2AX foci 24 h post-treatment. The alkaline comet assay also revealed the presence of DNA damage following melamine and CA co-exposure. *In vivo* assay also revealed the presence of melamine-CA complex in the kidney. These data indicated that the cytotoxic and genotoxic effects of melamine and CA co-exposure differ from those of melamine or CA alone.

## Introduction

Melamine is an organic nitrogenous compound that has a number of industrial uses, including the production of plastics, dyes, fertilizers, and fabrics. It was found to induce nephrotoxicity in both animals (1,2) and children (3) when it was illegally added to pet foods or milk powder. However, evidence from different studies suggested that melamine was relatively non-toxic (4), especially on kidney (5). Therefore, the nature of the nephrotoxicity of melamine remains to be a matter of controversy.

Cyanuric acid (CA), another thiazine contaminant found during the investigation, has similar effects as melamine, such as the induction of diuresis and bladder calculi at high doses with no indication of renal toxicity (6). It was suspected that the combined effects of melamine and CA might have played an important role in incidents in North America and China. Evidence supporting this conclusion came from animal models, where it was found that melamine and CA could form insoluble precipitate in renal tubules, and eventually lead to tubule blockage and degeneration (7,8). The melamine-CA complex is a highly ordered lattice structure of alternating melamine and CA molecules held together by multiple hydrogen bonds between each pair of molecules, which is insoluble in water (7). However, though the above-mentioned studies have clearly demonstrated the cytotoxic effects of the melamine-CA complex, studies on the joint action of melamine and CA are still scanty. Furthermore, since melamine was shown to exert mutagenic activity in rats (9,10), whether the co-exposure to melamine and CA exerts genotoxic damage needs to be clarified as well. This could be extremely important, as DNA damage, if not repaired properly, may cause problems in the long run.

To address these issues, in the present study, the cytotoxic effects of melamine-CA co-exposure in a human kidney 293 cells model were first evaluated using the 3-(4,5-dimethylthiazol-2-yl)-2,5-di-phenyltetrazolium bromide (MTT) assay. Subsequently, two sensitive methods to detect DNA damage, phosphorylated-H2AX (γH2AX) foci formation (11) and the comet assay (12), were used to evaluate the possible DNA damaging consequences of such exposure. In particular, since it has been speculated that the ratio and the total amount of CA and melamine ingested can contribute to renal intoxication (13,14), different combination ratios of melamine and CA co-exposure were examined. We focused on the joint action and mutagenic activity of melamine-CA co-exposure, trying to gain a better understanding of the nature of the nephrotoxicity of melamine and CA.

## Material and Methods

### Cell culture and reagents

Human embryonic kidney cells (293) were supplied by the Cell Bank of Type Culture Collection of the Chinese Academy of Science (China). The 293 cells were cultured in RPMI-1640 medium (Gibco Laboratories, USA) with 10% (v/v) fetal bovine serum. Melamine, CA, MTT, dimethylsulfoxide (DMSO), and 4,6-diamidino-2-phenylindole (DAPI) were purchased from Sigma (USA).

### Solution preparation and co-exposure

Melamine and CA (5 mg) were dissolved in 1 mL RPMI-1640 culture medium with 20 μL glycerol as a stock solution (5 mg/mL), with heating and stirring to assist dissolving. The final combined concentration for melamine and CA co-exposure was set at 0.5 mg/mL and the ratios between melamine and CA were set at 100:1, 10:1, 1:1, 1:10, and 1:100 (v:v). For example, for 100:1 co-exposure,1 mL melamine stock solution and 0.01 mL CA stock solution were added directly to a cell culture plate containing 9 mL culture medium. Other co-exposure treatments were similarly prepared. However, experiments using the 1:1 ratio of melamine and CA co-exposure were not performed, as strong precipitation occurred when 0.5 mL melamine and 0.5 mL CA were added to the cell culture plate.

### Cytotoxicity assay

The toxicity of melamine, CA, and their co-exposure on cells was examined using the MTT test. Briefly, cells were seeded into a 96-well culture plate at a density of 1×10^4^ cells/well. Twenty-four hours later, the medium was discarded and replaced with fresh medium containing 0.5, 1, or 5 mg/mL melamine or CA, or medium containing a final concentration of 0.5 mg/mL of 100:1, 10:1, 1:10, and 1:100 melamine-CA co-exposure. Cells were treated for 2, 12, or 24 h, and at the end of each time-point, 20 μL of MTT [5 mg/mL in phosphate-buffered saline (PBS)] was added to each well and further incubated for 3 h. Subsequently, the solution was discarded and 150 μL of isopropanol was added. After agitation for 10 min for the formazan to dissolve, absorbance at 570 nm was read on a microtiter plate reader (BioTek Instruments Inc., USA). Relative survival is reported as the absorbance of treated sample/absorbance of the control group.

### Immunofluorescent microscopy and quantification of γh2AX foci

Immunofluorescent microscopy was performed as described previously (15,16). Briefly, cells were seeded into a 6-well culture plate at a density of 1×10^5^ cells/well, with each well containing a glass coverslip. At the end of each treatment time-point, cells were fixed in 4% paraformaldehyde for 15 min, washed with PBS three times, and permeabilized in 0.2% Triton X-100. After treatment with blocking serum [goat serum (ThermoFisher Scientific, USA)] for 1 h, samples were incubated with mouse monoclonal anti-γH2AXantibody (1:3,000; Millipore, USA) at 4°C overnight, followed by Alexa Fluor 488‐conjugated goat anti‐mouse secondary antibody (1:300; Invitrogen, USA) for 1 h. DAPI was added to the cells to stain the nuclei and incubated at 37°C for another 15 min. Finally, the coverslip was removed from the plate, mounted onto a glass slide, and observed using a Leica MDI4000 fluorescent microscope (Leica, Germany).

To prevent bias in the selection of cells that exhibited foci, all cells were counted in the field of vision. Image‐Pro Plus (Media Cybernetics, USA) was used to count the γH2AXfoci in each cell. In addition, to exclude relatively weak foci and background spots, a setting was used as a standard for quantification in all cells selected for analysis (13).

### Alkaline comet assay

The alkaline comet assay was conducted as described earlier with certain modifications (15,16). Briefly, fully frosted microscope slides were first covered with 150 µL of 0.65% normal melting point agarose and immediately covered with a coverslip. Slides were placed on ice for 8 min to allow the agarose to solidify. Subsequently, the coverslips were removed and the first agarose layer was covered with the cell suspension (1×10^5^ cells in 15 µL PBS were mixed with 75 µL of 0.65% low melting point agarose). The coverslip was replaced and the slide was allowed to solidify on ice for 8 min. A third layer of agarose (90 µL of 0.65% low melting point agarose) was added as described above. Finally, the coverslips were removed and the slides were immersed in the lysis buffer [2 M NaCl, 30 mM EDTA, 10 mM Tris, with 1% Triton X-100 and 10% DMSO added just before use (pH 8.2-8.5)] for 1 h at 4°C. The slides were removed from the lysis buffer and transferred to an electrophoresis chamber. After equilibration in 0.5× TBE [Tris/boric acid/EDTA buffer (Bio-Rad, Hercules, USA)] for 20 min, electrophoresis was conducted at 60 V and 300 mA for 20 min. The slides were washed in a neutralization buffer [0.4 mM Tris (pH 7.5)] twice for 5 min. The slides were drained and stained with ethidium bromide and observed with a fluorescent microscope.

Single cell images were captured and analyzed using a Leica DMI4000 immunofluorescent microscope (Germany) and tail moment was measured by the Comet Assay Software Project Lab (http://casplab.com/index.php).

### Animal experiments

Four-week-old Institute of Cancer Research (ICR) mice were purchased from the China National Laboratory Animal Resource Center (China). The mice were kept in our animal facilities (illuminated with strip lights, 200 lx at cage level with a photoperiod of 12 h light/dark cycle; 22±1°C) for 1 week prior to the experiments. The rats were allocated randomly to each group using random numbers created by Microsoft Excel (USA). During the housing, the animals were monitored once daily for health status. All experimental protocols were approved by the Ethics Committee of Hangzhou Normal University and in accordance with the Guide for the Care and Use of Laboratory Animals (Ministry of Science and Technology of China, 2006). All efforts were made to minimize suffering.

### Nephrotoxicity of melamine-CA co-exposure *in vivo*

ICR mice were divided into control group, fed with standard mice chow diet, and dietary melamine-CA co-exposure group (experimental group), fed with chow diet containing 1% melamine and 0.1% cyanuric acid, with 10 mice in each group. The test chemicals in powder form were weighed using a designated analytical scale and dissolved in dissolved in double-distilled water. The solution with each test compound was thoroughly mixed with 100 g chow diet under a chemical hood and dried. This material served as “stock feed” and was stored under nitrogen at -20°C. The “stock feeds” were mixed with additional standard mice chow to the final dosing concentrations and aliquoted in 500 g lots and stored at −20°C until administration to animals within one week. The final concentrations for melamine and CA were 10 and 1 g/kg feed, respectively.

At the end of the experiment (5 weeks), all surviving mice were sacrificed and necropsied, and kidneys were photographed, weighed, and fixed in formalin (10%) for paraffin sections analysis.

### Histological examination

The formalin-fixed kidneys were embedded in paraffin after undergoing ethanol dehydration, and were then cut into 5-μm sections for H&E staining following standard protocols (15). The crystal complex in renal tubules of mice was examined by a reflection-mode cross-polarized microscope (XY-PRT, Sunny Optical Technology, China).

### Electron microscopy examination

The ultrastructure of calculi from kidneys of the mice were examined using a field-emission scanning electron microscope (FSEM, SIRION-100; FEI, USA) equipped with a GENESIS4000 energy dispersive X-ray spectroscope (EDS, EDAX-GENESIS4000, USA) microanalyzer operated at 25 kV and 280 μA (16). The measurements are reported in micrometers (μM).

### Statistical analysis

Statistical analysis was performed with Student's *t*‐test using SPSS 12.0 software (IBM, USA). Each experiment was conducted independently ≥4 times. Data are reported as means±SD. P<0.05 was considered to indicate a statistically significant difference.

## Results

### Cytotoxic effects of melamine, CA, and melamine‐CA co‐exposure

**Figure 1:**
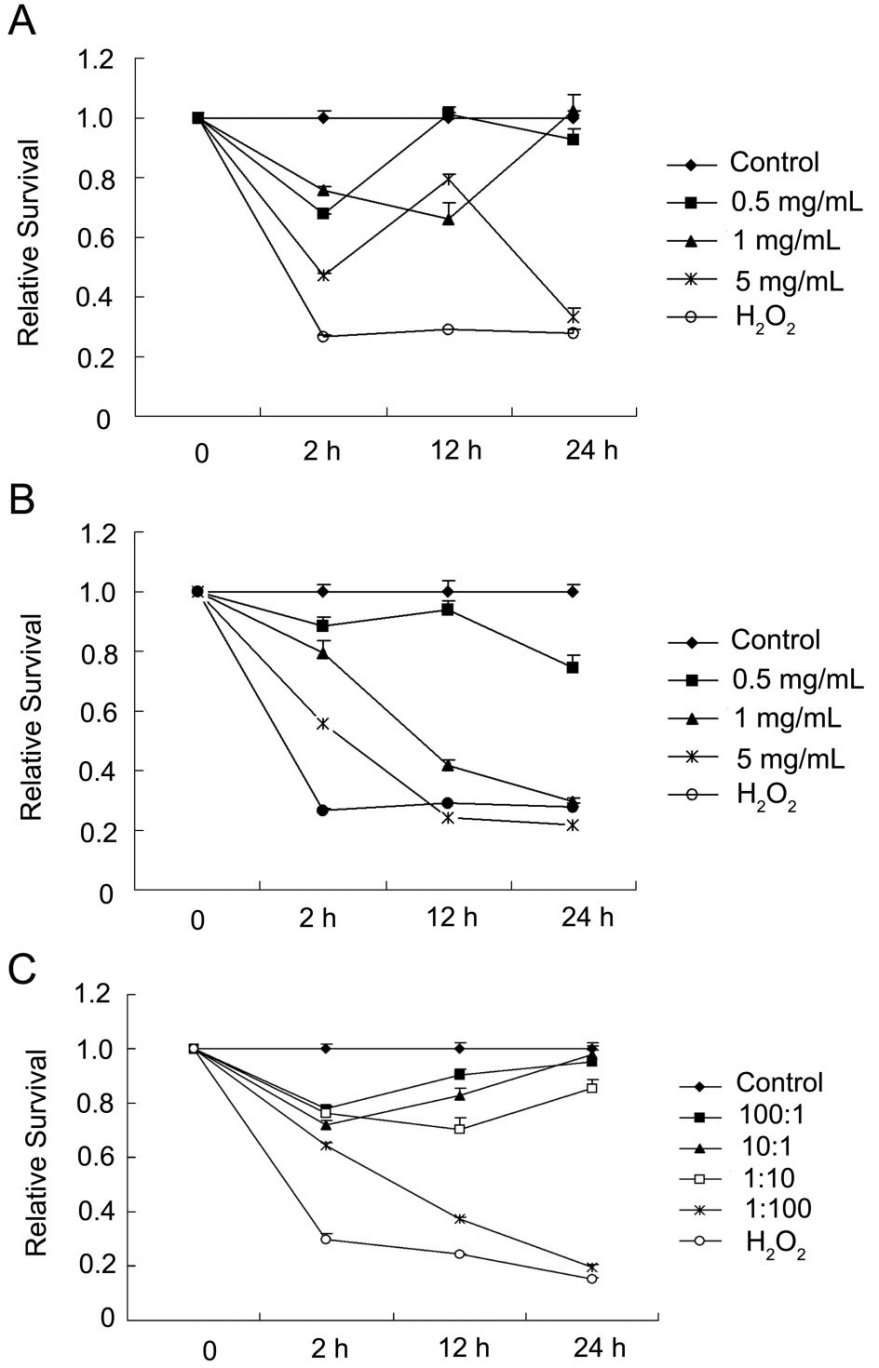
Cytotoxic effects of melamine, cyanuric acid (CA), and melamine and CA co-exposure on 293 cells. The 293 cells were treated with different concentrations of melamine, CA, and melamine-CA at different ratios (0.5 mg/mL) for ≤24 h and cell viability was measured by the MTT test. **A**, Melamine alone; (**B**) CA alone; and (**C**) melamine-CA co‐exposure. Data are reported as means±SD.

As shown in Figure 1A, no significant cytotoxicity was found for melamine at 2 and 12 h, and there was only a slight but significant decrease of cell survival at 24 h for all three concentrations. For CA at 0.5 mg/mL, as shown in Figure 1B, cytotoxicity was only observed at 24 h, while at higher concentrations CA led to a steady decrease in cell viability. Based on these results, 0.5 mg/mL was chosen as the final concentration for co-exposure analyses. As shown in Figure 1C, it was found that at the 100:1 and 10:1 ratios, when melamine was in excess, cell survival was decreased at 2 h, but recovered gradually at later time-points. Melamine-CA co‐exposure at a 1:10 ratio showed a similar pattern, but with stronger cytotoxicity; however, when CA was in excess (1:100), co-exposure steadily decreased cell survival and at 24 h, the survival rate was decreased to around 20%, almost the same as the H_2_O_2_ positive control. These data suggested that the different combinations of melamine-CA have distinct cytotoxic effects on 293 cells survival compared to melamine or CA alone, and that the higher the amount of CA, the stronger the cytotoxicity.

### Effects of melamine, CA, and melamine‐CA co‐exposure on γH2AX foci formation

**Figure 2:**
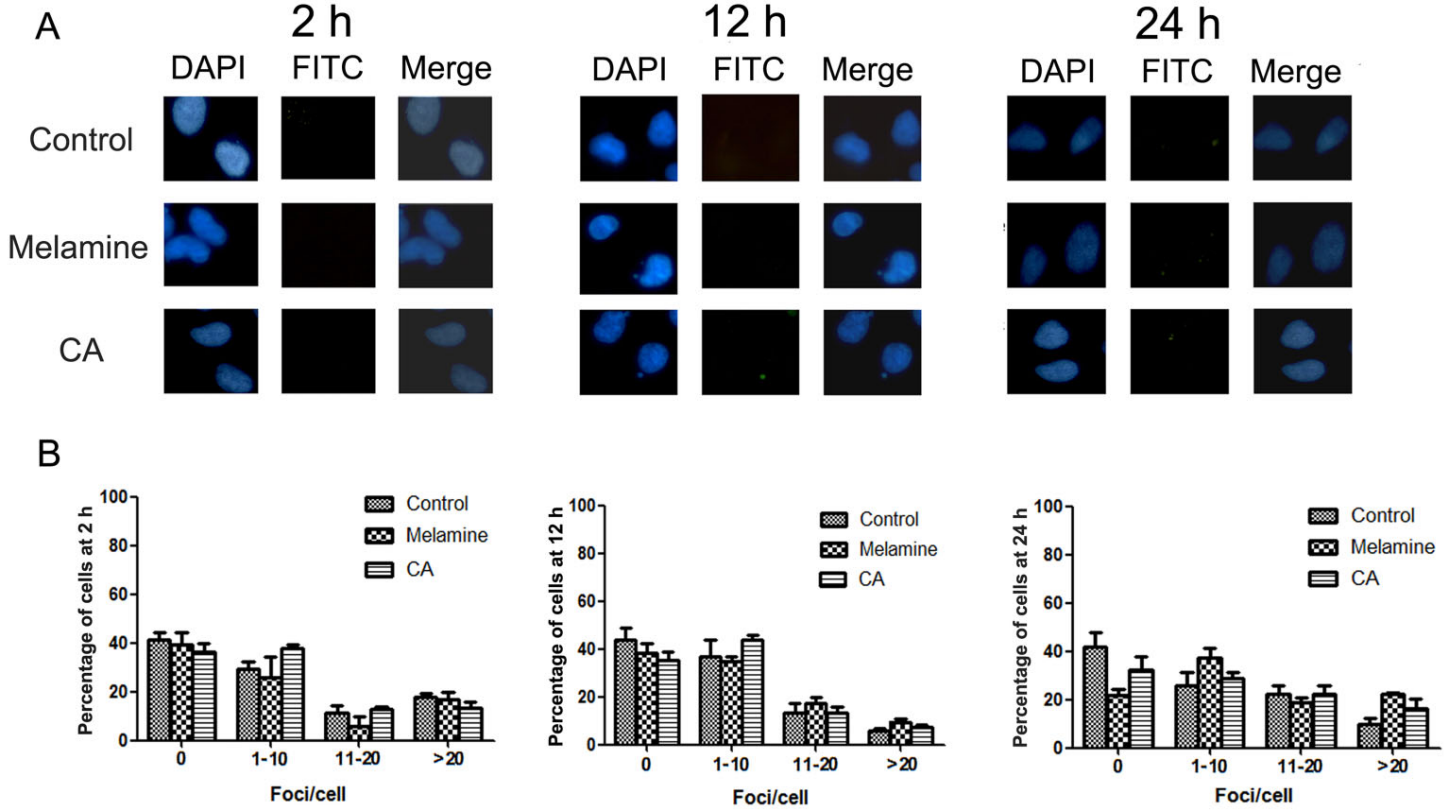
Neither melamine nor cyanuric acid (CA) alone induced phosphorylated-H2AX (γH2AX) foci formation in 293 cells. After melamine or CA treatment for 2, 12, and 24 h, cells were fixed and stained with anti‐γH2AX antibody and subjected to immunofluorescent microscopy. **A**, Representative images from 1 of 4 independent experiments. Upper panel, control; middle panel, melamine-treated cells; lower panel, CA-treated cells. Blue, 4,6‐diamidino‐2‐phenylindole (DAPI) stain for nuclei; fluorescein isothiocyanate (FITC), green stain for γH2AX. **B**, Semi‐quantification of γH2AX-positive cells from (**A**). Data are reported as means±SD.

Figure 2A shows representative immunofluorescent images of the 293 cells treated with 0.5 mg/mL melamine or CA and Figure 2B shows semi‐quantification of the percentages of cells where the number of foci/cell fell within the indicated categories. These results showed that in untreated cells, ∼30% of cells contained no foci, >40% had 1–10 foci/cell, 10–20% exhibited 11–20, and the remaining 10–20% contained >20. Although melamine and CA treatment caused certain variations in the percentages of cells exhibiting the indicated number of foci/cell at different time points, none of these variations were statistically significant.

**Figure 3:**
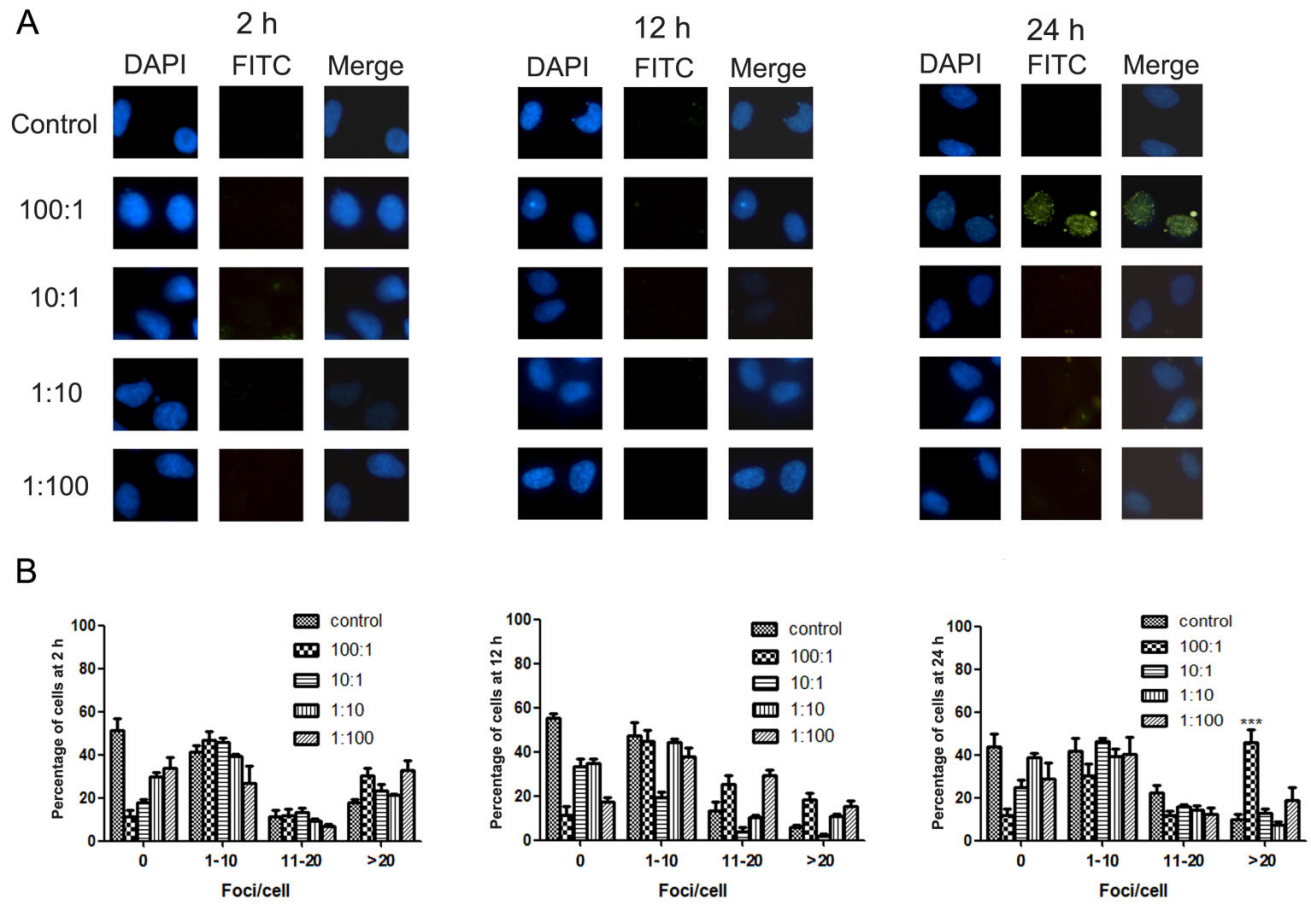
Effects of melamine-cyanuric acid (CA) co-exposure on phosphorylated-H2AX (γH2AX) foci formation in 293 cells. After melamine-CA treatment with various ratios of melamine-CA for 2, 12, and 24 h, cells were fixed and stained with anti‐γH2AX antibody and subjected to immunofluorescence microscopy. **A**, Representative images from 1 of 4 independent experiments. **B**, Semi‐quantification of γH2AX-positive cells from panel **A**. Data are reported as means±SD. ***P<0.001, compared to Control (ANOVA). DAPI, 4,6‐diamidino‐2‐phenylindole; FITC, fluorescein isothiocyanate.

Figure 3A shows the representative images of 293 cells treated with various ratios of melamine-CA for 2, 12, or 24 h and the semi‐quantification of these results is shown in Figure 3B. It was found that at ratios of 10:1, 1:10 or 1:100, the effects of melamine-CA co-exposure were similar to those observed following melamine or CA treatment alone. Such treatments also caused variations in the percentages of cells with the categorized number of foci/cell; however, there were no statistically significant differences between the treatment groups and controls. When cells were treated with melamine‐CA at a ratio of 100:1, no significant difference was found 2 or 12 h after treatment. However, this treatment significantly increased the percentage of cells with >20 foci/cell at 24 h, from ∼10% in the control group to <50% (Figure 3B). Therefore, it appears that in certain combinations, melamine-CA co-exposure can induce H2AX phosphorylation.

### Alkaline comet assay analysis

**Figure 4:**
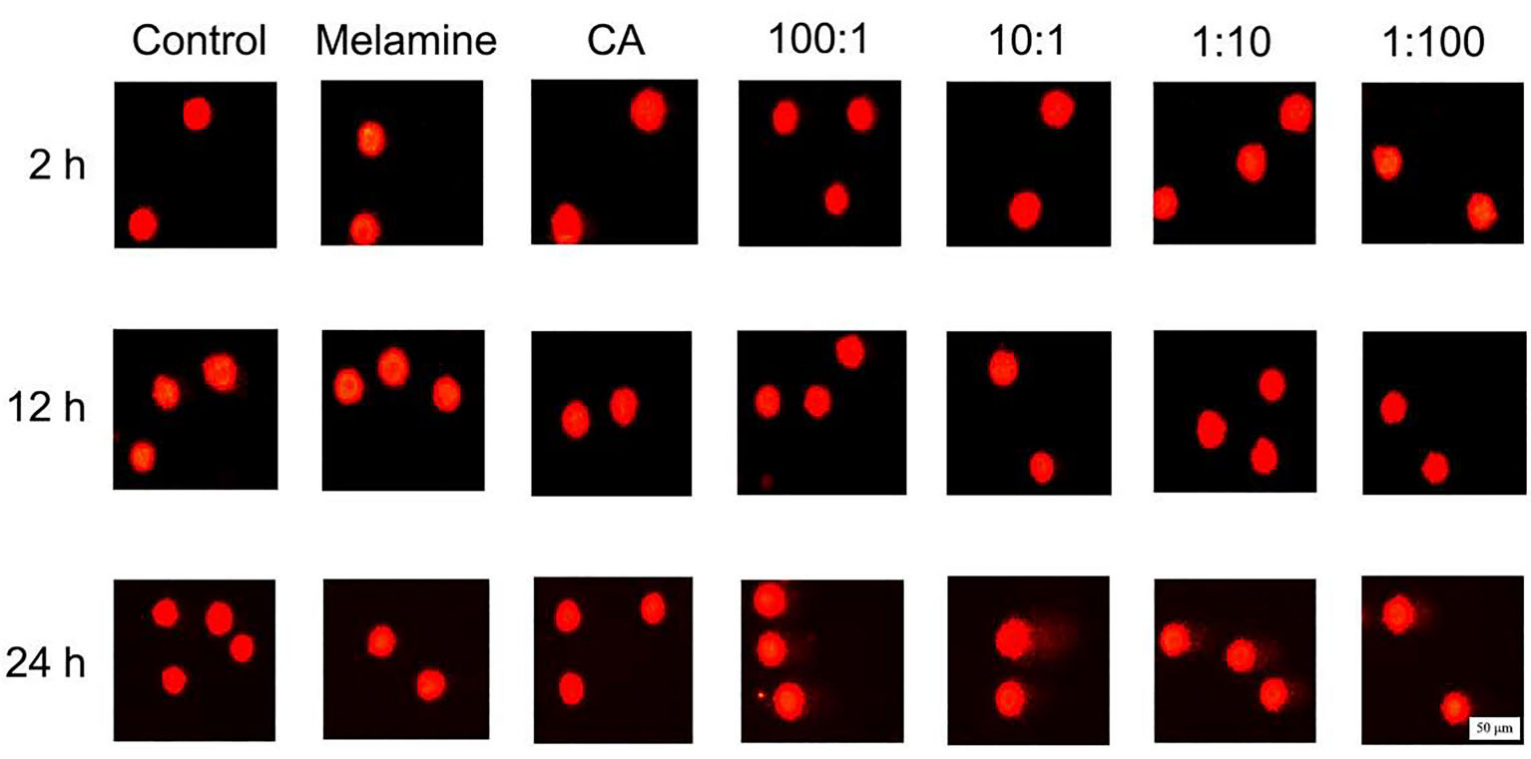
Alkaline comet assay of 293 cells treated with melamine, cyanuric acid (CA), or various ratios of melamine-CA. Representative images of cells at different time-points are shown. Scale bar: 50 μm.

Shown in Figure 4 are the representative images of the alkaline comet assay and the calculated tail moments are reported in Supplementary Table S1. In the untreated cells, tail moments ranged from 0.4-0.7 and neither melamine nor CA treatment alone caused any significant increase in tail moment compared to controls, consistent with previous studies that neither melamine nor CA is genotoxic. In contrast, while the 4 combinations of melamine-CA co-exposure did not affect the tail moment at 2 and 12 h, the tail moments were significantly increased at 24 h post-treatment, with an average tail moment >1.4. Thus, these data indicated that melamine-CA co-exposure can induce DNA damage.

### Renal effects of melamine-CA co-exposure

**Figure 5:**
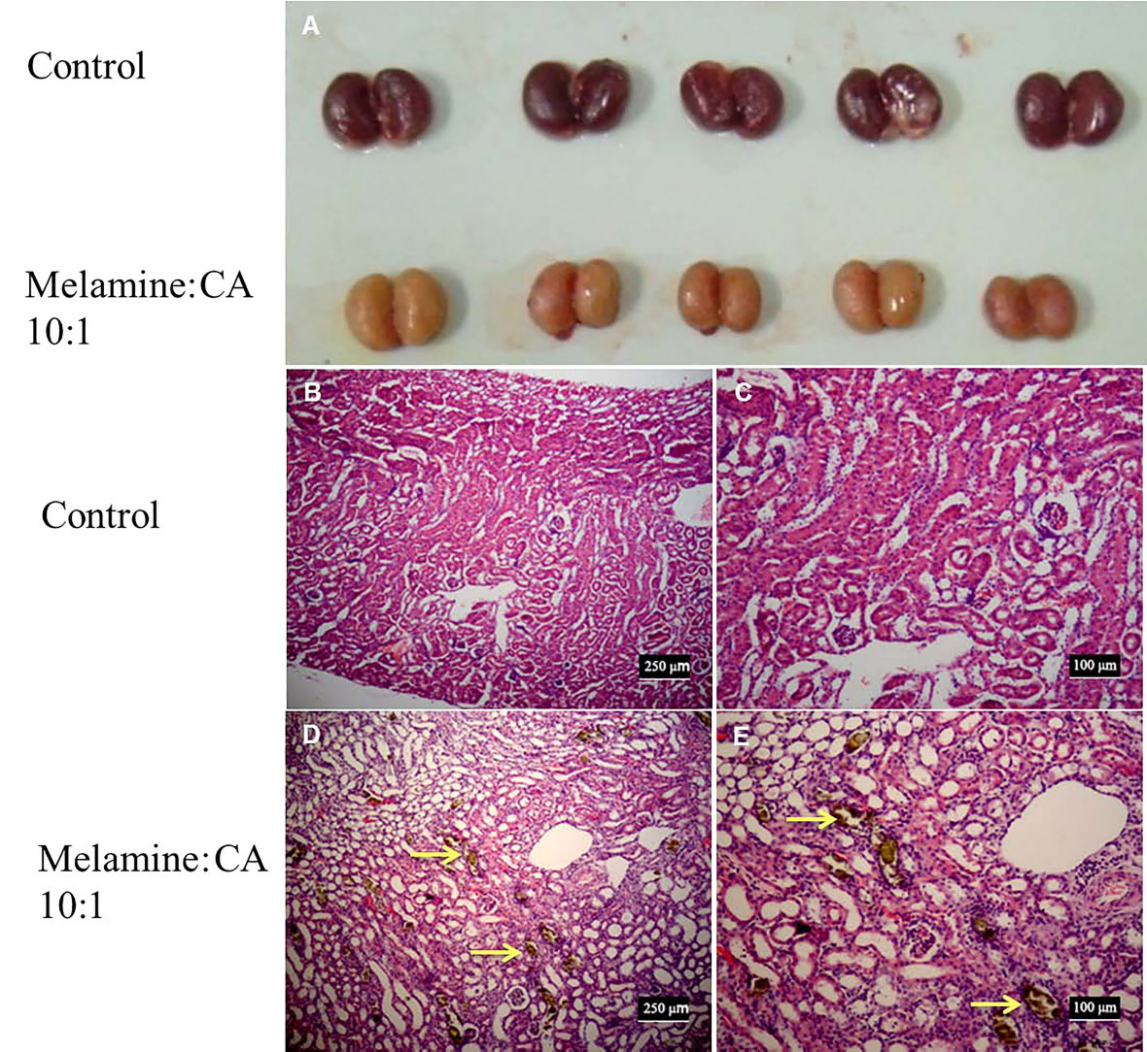
Effects of dietary co-exposure to melamine and cyanuric acid (CA) (10:1) on the morphological (**A**) and histological appearance of the kidney of mice. Representative photographs of histological examination for the 2 groups are shown in **B** to **E**. Golden-brown colored crystals (yellow arrow) were observed in renal tubules in the mice of experimental groups (**D** and **E**). Scale bars: 250 μm (B and D) and 100 μm (C and E).

**Figure 6:**
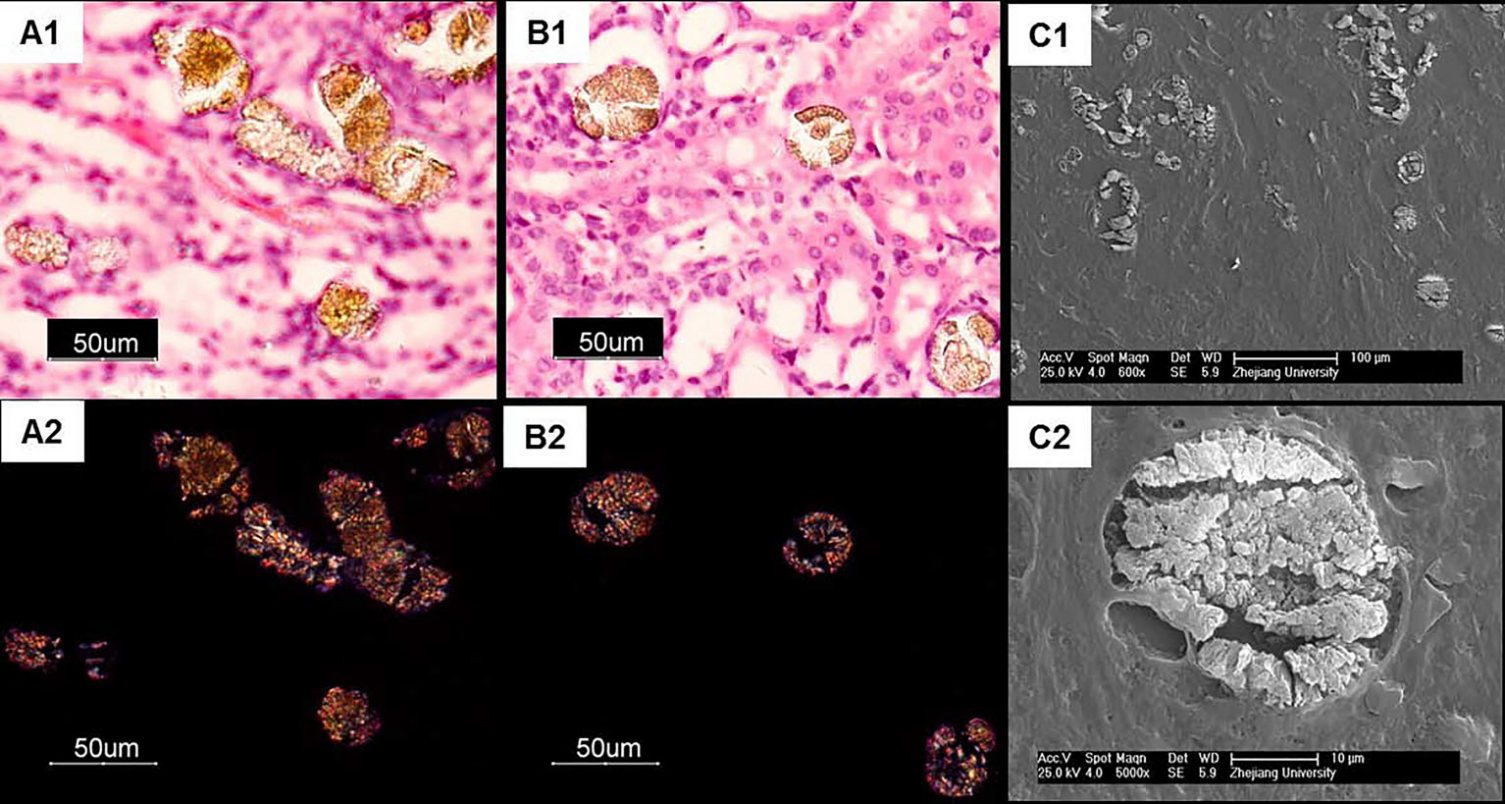
The crystal complex in the renal tubular lumina of the mice with dietary co-exposure to melamine and CA (10:1) examined by reflection-mode-cross-polarized microscope (**A1**, **A2**, **B1**, **B2,** scale bars: 50 μm) and field-emission scanning electron microscope (**C1**, **C2,** scale bars: 100 and 10 μm, respectively).

The effects of melamine plus CA on histological changes were assessed after 5 weeks of treatment. Morphological changes were observed in surviving mice with dietary co-exposure to melamine and CA (10:1) but not in the controls, including brownish color, enlarged size (Figure 5A), and increased kidney weight/body weight ratio (data not shown). Under the microscope, tubule-obstructed pathological changes were observed, and brownish needle-like crystals radially aggregated in tubular lumina were observed in the 2 groups (Figure 5D and E). The reflection-mode-cross-polarized microscope and field-emission scanning electron microscope examinations also revealed the formation of crystals in the tubular lumina of the mice with dietary co-exposure to melamine plus CA (10:1) (Figure 6).

## Discussion

The melamine scandal presents a classic example for a toxicological study: two relatively non-toxic substances, when acting together, become toxic. Indeed, humans are rarely exposed to only a single agent in isolation, but are much more likely to be exposed to a mixture of numerous environmental chemical pollutants. Therefore, not only is the mode(s) of action (MOA) of a particular pollutant dependent on the biological effects stemming from the mixture of its own set of metabolites, but the MOA can also be affected by co-exposure to pollutants that may have related or even unrelated targets or affected pathways (17) . Detailed studies have revealed that melamine and its derivative CA, when administered together, form insoluble crystals under certain conditions that eventually lead to damage of the tissues and organs in which these crystals reside (18,19). However, a relatively thorough understanding of the toxic effects of co-exposure to these two chemicals is lacking.

Since at 0.5 mg/mL melamine and CA showed only mild cytotoxic effects (Figure 1), this concentration was chosen as the final combined dose for co‐exposure to identify any possible toxic effects. Different combination ratios of the two chemicals were examined, as this could be another contributing factor for the toxic effects. We observed that when melamine and CA were added to the cell culture at a 1:1 ratio, precipitation occurred immediately and almost all cells died quickly within hours (data not shown). This severe cytotoxic effect of the precipitate may be the reason for the renal damage in affected animals, as such precipitates could be observed in kidney tubules (Figure 4), particularly in the medulla, even by the naked eye (7). Indeed, in nearly all studies conducted so far, melamine and CA have been combined in a 1:1 ratio, which led to acute nephrotoxicity or even death in experimental animals within days at high doses (20–23).

By contrast, it was found that when cells were exposed to these two chemicals at ratios of 100:1, 10:1 or 1:10 (melamine:CA, v:v), cell viability was only transiently affected; for example, cell viability decreased at 2 h and gradually recovered (Figure 1C). The transient cytotoxic effect could be the result of small amounts of precipitates formed during co-exposure, although other mechanisms cannot be ruled out. In contrast, melamine-CA co-exposure at a ratio of 1:100 exhibited distinct cytotoxic effects, with the cell survival rate decreasing steadily with time (Figure 1C). This cannot be explained solely by the formation of precipitates, as some level of precipitation would also be expected in one or more of the other three co-exposure conditions. Thus, the exact mechanism for the toxic effect at this specific mixture ratio requires further investigation. However, in the study by Chen et al. (24), in which contaminated dog food with a melamine:CA ratio of 6.8:1 was used, it was shown that intake of this diet for 12 weeks did not cause acute renal toxicity in rats. In addition, rats fed with ≤20% of such a diet for 3 months did not develop renal failure and renal toxicity, that only became evident at higher doses. Together with the present results, these data indicated that the mixture ratio between melamine and CA is an important factor in determining the cytotoxic effect of co-exposure to these two chemicals. Therefore, the ratio between melamine and CA requires further consideration in analyzing co-exposure effects in future studies.

γH2AX foci formation has now been accepted as a sensitive method for detecting DNA damage (25,26). Using this method, it was found that neither melamine nor CA alone induced significant γH2AX foci formation at 0.5 mg/mL (Figure 2), and no genotoxicity, which is consistent with previous observations (27). Of note, a significant increase in γH2AX foci formation was observed in cells exposed to melamine-CA at a 100:1 ratio at 24 h (Figure 3). By contrast, the alkaline comet assay results indicated that DNA damage could be induced by all four co-exposure conditions, but not by melamine or CA alone (Figure 4). Although the γH2AX foci assay and comet assay results were not completely consistent, considering the difference in the underlying mechanisms for the two assays, such inconsistency is not unexpected (11). In any case, the two assays detected DNA damage in the presence of the two compounds, but not in the presence of either compound alone. Ke et al. (28) examined the urinary level of 8‐hydroxy‐2'‐deoxyguanosine (8-OHdG) in infants fed with the melamine-contaminated powdered formula and found no significant differences in the mean urinary 8-OHdg concentrations between these infants and the control groups; therefore, it was concluded that melamine may not have caused any increase in oxidative damage of DNA in infants. There may be several possible explanations for these contradictory results. One may be associated with the different detection methods for DNA damage (such as γH2AX foci formation and the comet assay). Another possible reason is that the observed DNA damage in the present experimental setting may be repaired at later time-points after 24 h by the cellular DNA repair system. The cell culture model used is also quite different from the human body and therefore, the response to melamine-CA co-exposure may be extremely different between the cell model and an entire organism. There might also be other explanations for these contradictory genotoxic results. Thus, a more detailed study is required to answer this question.

Melamine and CA co-exposure exhibited significant nephrotoxicity *in vivo* at the ratios of 10:1. The two agents together provoked a pattern of renal injury that resembled clinical findings described in patients. These findings were supported by the results of the *in vitro* experiment in our study, further supporting the idea that the mixture ratio between melamine and CA might be an important determinant of the cytotoxic effect of co-exposure to these two chemicals.

In summary, melamine and CA co-exposure exhibited different cytotoxic effects compared to melamine and CA alone, and the combination ratio could be a determining factor for the cytotoxic effect. In addition, melamine and CA co-exposure also induced DNA damage in 293 cells; however, this requires further verification by different cell types and *in vivo* animal models.

## Supplementary Material

Click here to view [pdf].
